# Cervical Lateral Mass and Pedicle Fracture Reduced with a Herbert Screw: A Technical Note

**DOI:** 10.3390/medsci13030092

**Published:** 2025-07-19

**Authors:** Antonio Colamaria, Francesco Carbone, Augusto Leone, Giuseppe Palmieri, Savino Iodice, Bianca Maria Baldassarre, Giovanni Cirrottola, Valeria Ble, Uwe Spetzger, Giuseppe Di Perna

**Affiliations:** 1 Division of Neurosurgery, Policlinico “Riuniti” Foggia, University of Foggia, 71122 Foggia, Italy; colamariaa@gmail.com (A.C.); francesco.carbone615@gmail.com (F.C.); guseppepalmierifdoc@gmail.com (G.P.); savio.tesi@libero.it (S.I.); baldassarrebianca@gmail.com (B.M.B.); cirrog@hotmail.com (G.C.); valeryblu13@gmail.com (V.B.); uwe.spetzger@klinikum-karlsruhe.de (U.S.); dr.giuseppediperna@gmail.com (G.D.P.); 2 Department of Neurosurgery, Karlsruher Neurozentrum, Städtisches Klinikum Karlsruhe, 76133 Karlsruhe, Germany; 3 Faculty of Human Medicine, Charité Universitätsmedizin, 10117 Berlin, Germany

**Keywords:** cervical fracture, spinal surgery, precision medicine, Herbert screw

## Abstract

Background: Traumatic fractures of the cervical spine pose significant challenges in management, particularly in young patients, where preserving mobility is crucial. Patient Characteristics: A 30-year-old woman presented with a C3 lateral mass and pedicle fracture following a motor vehicle collision. Initial conservative management with a rigid cervical collar for three months failed to reduce the diastasis, and the debilitating neck pain worsened. Preoperative imaging confirmed fracture instability without spinal cord compression. Intervention and Outcome: Preoperative screw trajectory planning was conducted with the My Spine MC system (Medacta), and fine-tuning was achieved on a 3D-printed model of the vertebra. A posterior midline approach was employed to expose the C3 vertebra, and a Herbert screw was inserted under fluoroscopic guidance. Imaging at three months demonstrated significant fracture reduction and early bone fusion. The patient achieved substantial improvement in functional mobility without complications. Conclusion: Herbert screw fixation holds potential as a less-invasive alternative to conventional posterior stabilization for selected cervical fractures. This technical note provides the reader with the required information to support surgical planning and execution.

## 1. Introduction

Subaxial traumatic cervical facet fractures represent a complex issue and often require personalized surgical management, thus remaining a topic of debate. Traditional posterior stabilization techniques are effective in cases of instability but inevitably result in reduced cervical mobility and potential long-term functional limitations. Advances in surgical technology and techniques provide the opportunity to refine these approaches, shifting the focus toward less-invasive and patient-specific solutions in the precision medicine era. In this technical note, the use of a Herbert screw for the anatomical reduction of a unilateral C3 lateral mass and pedicle fracture is described. Despite this technique deviating from conventional posterior stabilization methods, it allowed for the less-invasive, tailored stabilization of the injury while avoiding the long-term risks and disadvantages of a multilevel fixation. The choice to utilize this approach was driven by the young age of the patient and the isolated lateral mass and pedicle combined isolated fracture without vertebral artery injury, and following extensive discussion with the patient regarding surgical and conservative treatment options. Through the employment of the My Spine MC system, precision in screw placement was ensured. This case underscores the potential of combining innovative fixation techniques with advanced preoperative planning tools to achieve optimal clinical outcomes.

## 2. Materials and Methods

**Figure 1:**
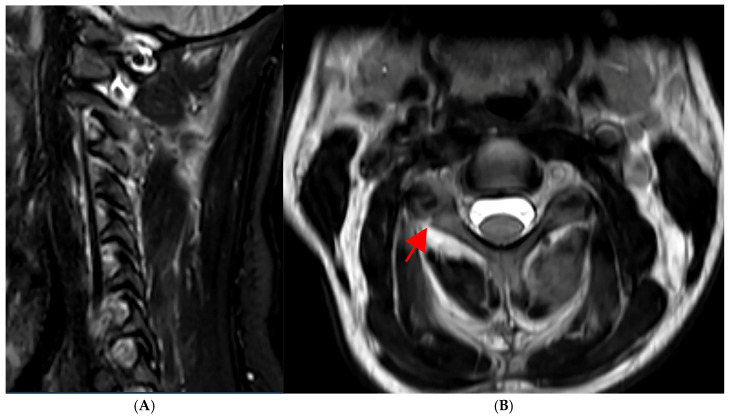
(**A**,**B**): MRI scan after emergency admission following a motor vehicle accident. (**A**) Sagittal FLAIR- and (**B**) axial T2-weighted scans documenting a diastatic fracture of the right facet and pedicle of C3 (AO Spine C3, F3, N0) without spinal compression (red arrow).

A 30-year-old woman presented with isolated neck pain after a motor vehicle collision resulting in a right C3 lateral mass and pedicle fracture (AO Spine C3, F3, N0) (Figure 1A,B). No signs of transverse foramen breach with vertebral artery involvement were present in the radiological examination (e.g., intraforaminal hematoma, vessel asymmetry or signal voids); therefore, a CT Angiography was waived due to the patient’s concerns regarding administered contrast.

**Figure 2:**
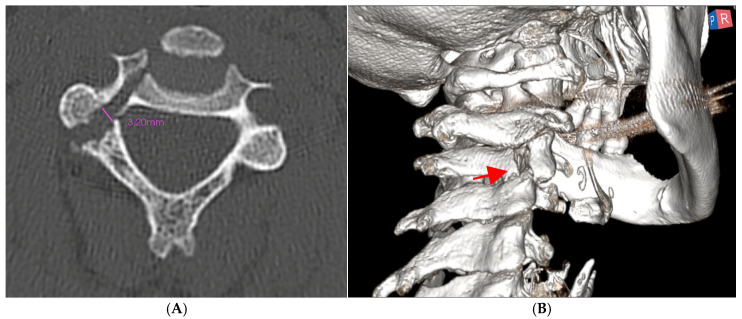
(**A**) axial and (**B**) 3D reconstruction of CT scans following 3 months of conservative treatment with a rigid collar. The progression of the distance between the heads of the fractures prompted the indication for surgical treatment. The red arrow indicates the fracture line.

A formal neurological examination did not reveal focal deficits, and after counseling, the patient decided to undergo first-line conservative management. The possibility of an external halo fixation was discussed, but this was eventually not chosen due to the patient’s concerns about potential side effects or discomfort [1,2]. After three months of rigid neck collar use, follow-up imaging (Figure 2A,B) showed progressive diastasis between the fracture fragments (3.02 mm), correlating with the reported persistent, activity-limiting neck pain with a Neck Disability Index (NDI) of 45% and a Numeric Rating Scale (NRS) of pain of 8.

Given these findings and the patient’s impaired quality of life, surgical intervention was deemed necessary and informed consent was obtained in written form. This study was conducted in accordance with the Declaration of Helsinki. A formal decision from an Ethical Committee was waived due to the previously obtained informed consent.

### 2.1. Preoperative Planning

**Figure 3:**
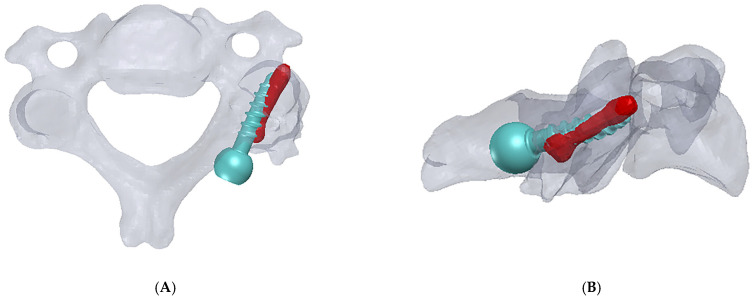

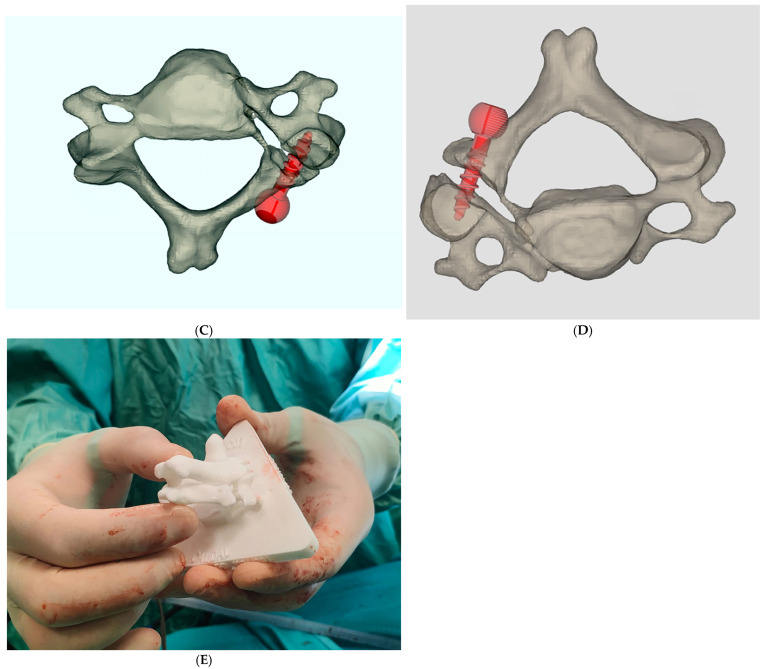
(**A**,**B**) The planning phase involved a detailed assessment of the vertebral anatomy and fracture configuration, enabling precise preoperative modeling and simulation. The green screw represents the originally planned trajectory, while the red one indicates the actual postoperative position. (**C**,**D**) Virtual screw placement trajectory was modeled in MySpine (Medacta, Castel San Pietro, Switzerland). (**E**) A 3D-printed vertebra with a planned entry point and trajectory was used as a model to match the patient’s vertebra (EOS 3D Printer [EOS, Munich, Germany], SLS printing technology; material: PA12).

A patient-specific surgical aid was created using the My Spine MC system (Medacta International, Castel San Pietro, Switzerland) to optimize the Herbert screw’s trajectory and placement (Figure 3A–E). This system is designed to help surgeons in preoperative planning, allowing 3D visualization based on the patient’s individual anatomy. This planning phase consisted of a detailed assessment of the vertebral anatomy and fracture configuration, enabling precise preoperative modeling and simulation. The ability to create a 3D rendering of the fractured vertebra was crucial for different reasons: firstly, it aided the planning of the trajectory in an abnormal anatomy; secondly, the rendering allowed us to specifically design the trajectory of the screw with which we were not familiar; and lastly, the mentioned software allowed us to create a 3D-printed model of the fractured vertebra that was used for preoperative insertion point and trajectory simulation.

### 2.2. Surgical Technique

**Figure 4:**
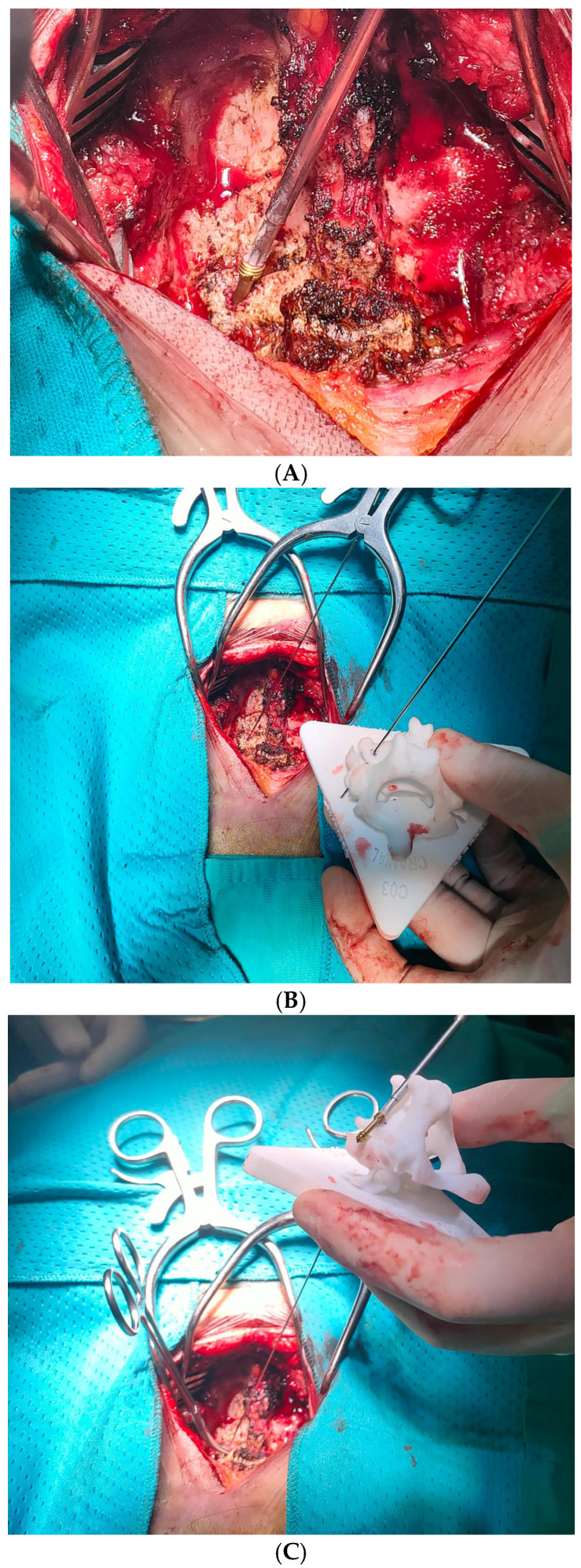

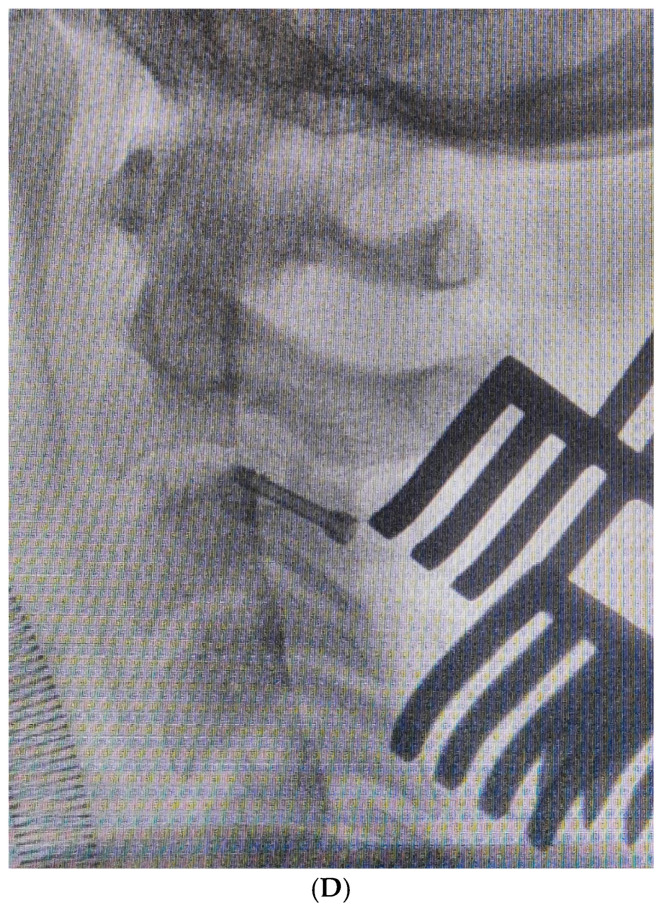
(**A**,**B**) The trajectory of Herbert’s screw is planned on the 3D-printed vertebra. (**C**) Direct visualization of the insertion point for Herbert’s screw and strict immobilization of the lateral mass were essential to prevent accidental injury to the vertebral artery. (**D**) Intraoperative fluoroscopy confirmed correct positioning.

Under general anesthesia, the patient was placed prone with her cervical spine immobilized to maintain stability. A posterior midline approach exposed the posterior aspect of the C3 vertebra while minimizing tissue disruption. Through the use of the custom guide generated by the My Spine MC system, a Herbert screw was inserted into the fracture site with meticulous attention given to anatomical reduction and proper screw alignment (Figure 4A–C). Intraoperative fluoroscopy confirmed correct positioning (Figure 4D).

Throughout the procedure, careful dissection and retraction techniques safeguarded adjacent neurovascular structures. To assure that the insertion of the screw would not further dislocate the fracture, potentially causing vertebral artery dissection, we minimized the movement of the lateral mass with a dissector posed in the lateroventral margin of it. The incision was then closed in layers, and the patient was fitted with a soft cervical collar postoperatively to facilitate healing and reduce mechanical stress on the fixation.

## 3. Results

**Figure 5:**
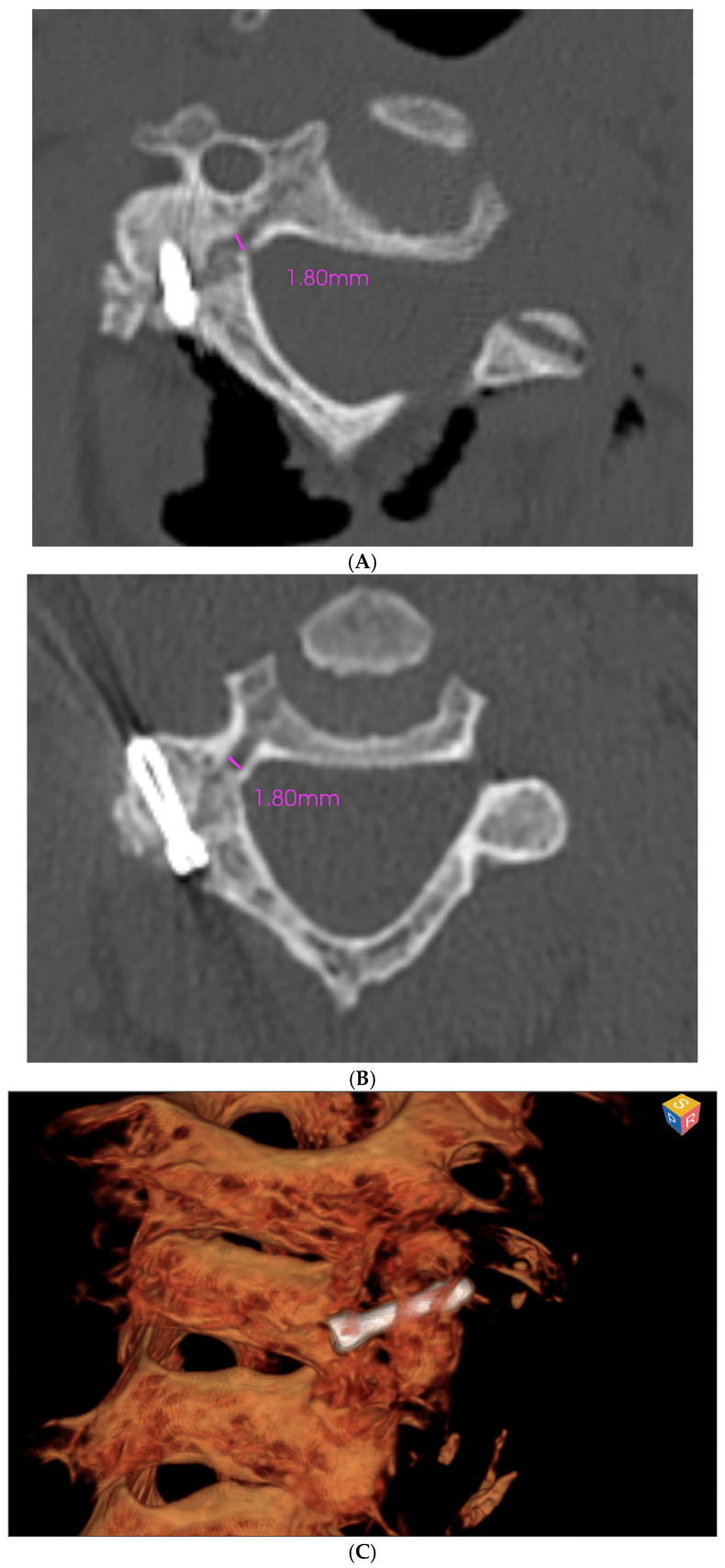
(**A**) Immediate postoperative scan highlighting fracture diastasis reduction and correct alignment of the bone heads as well as of the Herbert screw without violation of the vertebral artery canal. (**B**,**C**) CT scan and 3D reconstruction 3 months after the procedure, documenting bone fusion.

The patient’s recovery was uneventful, with immediate resolution of neck pain following surgery. Postoperative imaging confirmed proper alignment of the fracture fragments (1.8 mm diastasis, compared to 3.2 mm preoperatively) and stable fixation (Figure 5A).

The patient was discharged three days following the procedure with a six-week soft cervical collar regimen and a structured rehabilitation protocol focusing on gradual mobilization. Bisphosphonates (neridronic acid 25 mg/day for 16 days) were administered to support bone healing.

At the three-month clinical follow-up, the patient reported significant improvement in functional status (NDI of 14% and NRS of 2/10) and neck mobility, with complete resolution of pain. Preoperative and postoperative CT scans were compared using MySolution software (Medacta, Castel San Pietro, Switzerland, MySolution Ver 1.0), confirming accurate alignment and integration of the hardware (Figure 5B,C), further showing the reduction of the fracture from 3.2 mm to 1.8 mm as well as a sufficient distance from the transverse foramen. This software is specifically designed for joint replacement surgery, allowing pre- and postoperative 3D visualization of the implanted devices. No complications—such as wound infection, hardware displacement or neurological deficits—were observed.

## 4. Discussion

The present technical note highlights the benefits of adopting a Herbert screw for internal fixation of a traumatic, unilateral combined lateral mass and pedicle C3 fracture without vertebral artery injury. The described fracture represents an ideal indication for surgical management due to the intrinsic high instability and possible catastrophic consequences of further dislocation. Nonetheless, due to the reticence of the patient to undergo dorsal stabilization, we had to proceed with conservative treatment, with a cervical collar, as a Halo vest was also not accepted.

Facet dislocations in the subaxial cervical spine (C3–C7) account for about 10% of all injuries in this region, and approximately 40% of these cases are associated with neurological deficits [3]. Primary surgical treatment should be extensively discussed within counseling if the fracture is displaced or if the patient presents neurological deficits. These injuries have been classically addressed with posterior approaches, even though some authors advocate for an anterior-alone surgical treatment [4]. Non-displaced or minimally displaced fractures might be treated conservatively [5]. If this management method is chosen, serial radiographical follow-ups should be performed to identify patients whose fracture is not correctly healing [5].

The initial paucisymptomatic presentation of the patient and her young age prompted a conservative treatment following international standards [6]. Nevertheless, radiological evidence of progressive diastasis after three months and worsening axial neck pain suggested the need for cervical stabilization, which would have required C2–C4 dorsal spondylodesis. Considering the significant reduction in range of motion (ROM) that the 30-year-old patient would have experienced following the procedure, we decided to adopt an internal lateral facet and pedicle fixation following intraoperative approximation of the fractured poles using a Herbert screw. This screw-based internal fixation provides stable fracture compression in small bones, particularly the scaphoid and mandible [7,8]. Its headless design reduces soft-tissue irritation, aids precise fragment alignment, and allows early mobilization [9]. Clinical studies show reliable fracture healing, low complication rates, and positive functional outcomes, also in pediatric patients [10].

In this case of AO Spine C3 F3 N0 fracture, the disruption of the pedicle and lamina resulted in the disconnection of superior and inferior articular processes at the C3 level, leading to the instability and progressive distraction of the fractured heads under conservative treatment. As previously indicated by Cirillo et al. [11], lateral floating mass fractures have 5.41 times higher odds of failure of nonoperative management than other less complex facet fractures, as seen in the described case. Nevertheless, one of the primary challenges in the decision-making process for the management of so-called F2 and F3 fractures is determining the presence of mechanical instability, which remains a topic of debate [12,13].

The described internal fixation technique offers several advantages over conventional posterior stabilization, including the preservation of cervical mobility and reduced perioperative morbidity as well as minimal tissue disruption, shorter operative times, and, potentially, a shorter length of stay. The integration of the My Spine MC system enabled precise preoperative planning and intraoperative execution, highlighting the transformative potential of personalized surgical aids in complex spinal procedures.

While this approach demonstrated favorable outcomes, its broader applicability warrants further investigation. In the presented case, a “standard” posterior stabilization would have required an at least two-level posterior instrumentation in the young patient, which would have significantly reduced head mobility and increased the risk of iatrogenic neurovascular damage. Although we acknowledge that the presented technique cannot be implemented blindly in cases of isolated cervical posterior and middle column fractures, we do believe that its adoption may be motivated in specific (semi)-elective cases, where the planning of the insertion point and trajectory is possible. The pre- and postoperative alignment evaluation was primarily based on visual inspection using MySolution software and manual measurement of fracture diastasis. While this approach showed satisfactory anatomical reduction, it lacks standardized quantitative metrics or validated registration protocols. Therefore, the absence of objective 3D validation represents a limitation of the current study. Future implementations should consider incorporating image registration tools and quantifiable parameters (e.g., angular deviation, translation measurements) to ensure reproducibility and objective alignment verification. Moreover, to date, only cases of odontoid anterior fixation have been reported using a Herbert screw in spinal surgery, yielding good functional and fusion outcomes [14,15]. However, concerning its use in subaxial segments, long-term studies are needed to evaluate the durability of fixation, potential impacts on adjacent segments, and comparative efficacy relative to established techniques. Additionally, the cost-effectiveness and learning curve associated with implementing patient-specific technologies in routine practice merit exploration.

## 5. Conclusions

Herbert screw fixation holds potential as an alternative to conventional posterior stabilization in the management of selected isolated cervical spine fractures, particularly in young patients where the preservation of cervical mobility is a key concern. The approach described may achieve stable internal fixation while avoiding the morbidity associated with multilevel fusion. The use of patient-specific surgical planning tools further enhances the precision of screw placement and contributes to favorable outcomes. This technical note outlines the critical steps of the preoperative planning and surgical technique, providing clinicians with practical guidance to support clinical decision-making and execution in similar cases. Further studies with larger cohorts and longer follow-up periods are warranted to validate the long-term efficacy, safety, and biomechanical stability of this approach.

## Data Availability

The data provided in this study has been blinded for privacy reasons following the informed written consent of the patient. Nevertheless, additional data may be provided by the corresponding author upon reasonable request.
